# Beyond intracranial pressure: optimization of cerebral blood flow, oxygen, and substrate delivery after traumatic brain injury

**DOI:** 10.1186/2110-5820-3-23

**Published:** 2013-07-10

**Authors:** Pierre Bouzat, Nathalie Sala, Jean-François Payen, Mauro Oddo

**Affiliations:** 1 Department of Intensive Care Medicine, CHUV-University Hospital, Rue du Bugnon 46, BH 08.623, CH-1011 Lausanne, Switzerland; 2 Faculty of Biology and Medicine, University of Lausanne, Lausanne, Switzerland; 3 Joseph Fourier University, Grenoble, France; 4 Department of Anesthesiology and Intensive Care Medicine, University Hospital, Grenoble, France

**Keywords:** Traumatic brain injury, Cerebral blood flow, Brain oxygen, Cerebral metabolism, Multimodal monitoring, Cerebral microdialysis, Transcranial Doppler, Neuromonitoring

## Abstract

Monitoring and management of intracranial pressure (ICP) and cerebral perfusion pressure (CPP) is a standard of care after traumatic brain injury (TBI). However, the pathophysiology of so-called secondary brain injury, i.e., the cascade of potentially deleterious events that occur in the early phase following initial cerebral insult—after TBI, is complex, involving a subtle interplay between cerebral blood flow (CBF), oxygen delivery and utilization, and supply of main cerebral energy substrates (glucose) to the injured brain. Regulation of this interplay depends on the type of injury and may vary individually and over time. In this setting, patient management can be a challenging task, where *standard* ICP/CPP monitoring may become insufficient to prevent secondary brain injury. Growing clinical evidence demonstrates that so-called *multimodal* brain monitoring, including brain tissue oxygen (PbtO_2_), cerebral microdialysis and transcranial Doppler among others, might help to optimize CBF and the delivery of oxygen/energy substrate at the bedside, thereby improving the management of secondary brain injury. Looking beyond ICP and CPP, and applying a multimodal therapeutic approach for the optimization of CBF, oxygen delivery, and brain energy supply may eventually improve overall care of patients with head injury. This review summarizes some of the important pathophysiological determinants of secondary cerebral damage after TBI and discusses novel approaches to optimize CBF and provide adequate oxygen and energy supply to the injured brain using multimodal brain monitoring.

## Review

### Introduction

**Figure 1:**
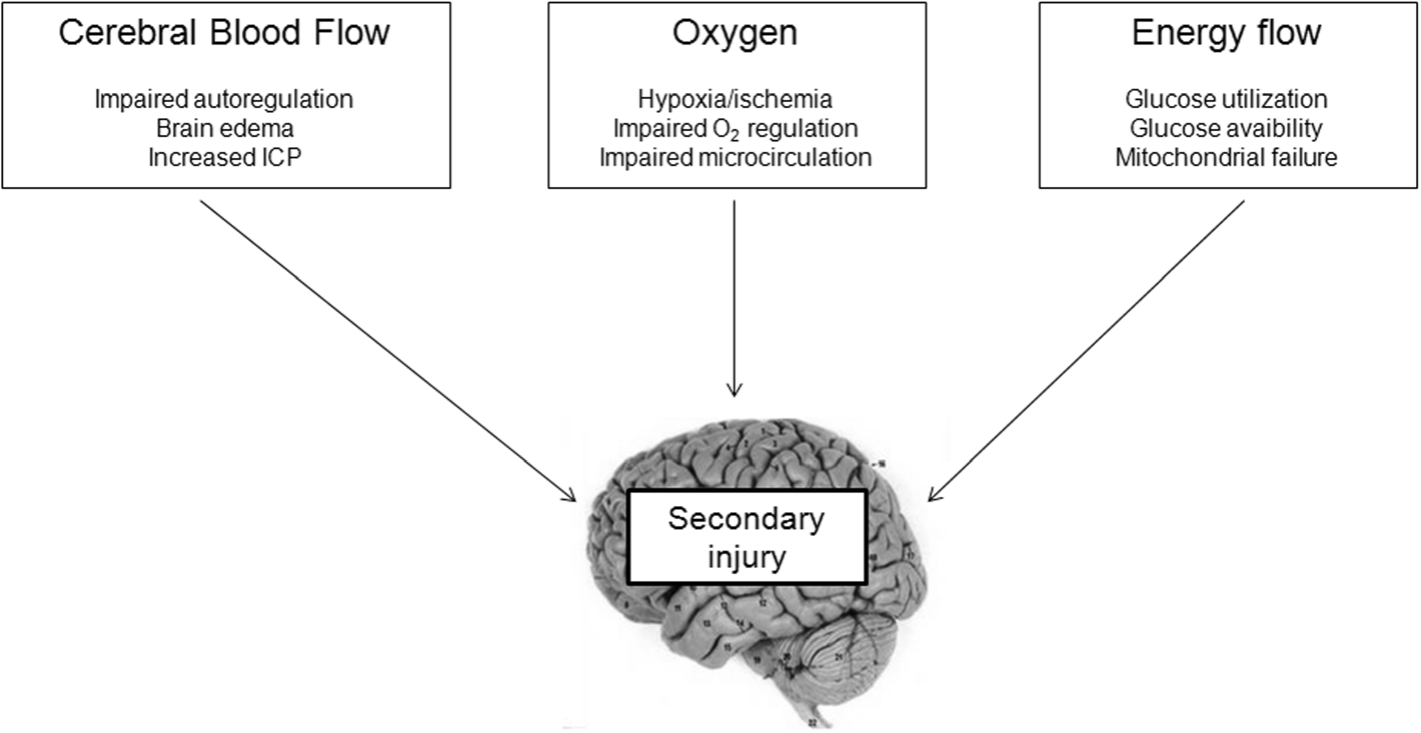
**Pathophysiology of secondary cerebral damage after TBI.** A schematic view of the pathophysiology of secondary cerebral damage after traumatic brain injury (TBI) that supports the concept of optimizing cerebral blood flow, the delivery of oxygen and the adequate supply of energy substrates.

Traumatic brain injury (TBI) first causes primary cerebral lesions related to the initial traumatic brain insult itself. In the early phase following TBI, a complex series of pathologic events triggers the propagation of a “secondary” injury cascade to cerebral areas initially not involved by TBI. Ischemia, hypoxia, and energy dysfunction are important determinants of secondary brain injury. Supporting the injured brain with adequate cerebral blood flow (CBF) and delivery of oxygen and energy substrate therefore is a mainstay of therapy after TBI (Figure 1). Despite this notion and the growing knowledge of posttraumatic secondary brain injury, the management of patients with TBI remains mainly focused to standard intracranial pressure (ICP)/cerebral perfusion pressure (CPP) therapy. Although this approach still constitutes an important part of TBI management [1,2], ICP-based monitoring and treatment alone may not be enough to modify TBI prognosis [3]. This may be partly due to the complexity of TBI pathophysiology and the heterogeneity of TBI lesions [4]. Increasing clinical evidence suggests that multimodal brain monitoring, including brain tissue oxygen tension (PbtO_2_), cerebral microdialysis (CMD), transcranial Doppler (TCD) among others, may optimize CBF and the delivery of oxygen and energy substrates to the injured brain in individual patients. We defined neuromonitor as a monitoring device (invasive or noninvasive) that allows assessment of dynamic changes of cerebral physiology. Based on that, we restricted neuromonitoring techniques described in this review to those monitors allowing measurement of CBF, cerebral oxygenation, and energy substrates at the bedside in the ICU.

## Pathophysiology and diagnosis

### Macrovascular dysfunction

Under physiological conditions, the relationship between CBF and CPP is linked to the cerebral autoregulatory capacity, thus CBF remains constant over a wide range of CPP. After TBI, depending on the nature of the lesion (diffuse vs. focal), there may be a large heterogeneity in brain autoregulatory capacities [5]. Reduction of cerebrovascular reserve and impairment of cerebral autoregulation cause CBF to become increasingly dependent on CPP, thus CBF may be inadequate/insufficient despite CPP is within so-called “normal” ranges (50–70 mmHg) [6]. In addition, apart from the individual relationship between CBF and CPP, secondary elevations of ICP also could contribute to further decrease CPP and aggravate ischemia.

The decrease of CBF after TBI was well documented by different studies using xenon-enhanced computed tomography (CT) [5] or positron emission tomography (PET) [7] that revealed heterogeneous regional disturbance of CBF. It also is important to stress the point that reduced CBF does not necessarily mean ischemia. Rather, the balance between CBF and cerebral metabolic rate of oxygen (CMRO_2_) determines whether the tissue is ischemic or not. For example, the decrease of CBF can be matched by a decrease of cerebral metabolic rate of oxygen (CMRO_2_), implying adequate CBF and preserved metabolic coupling, as it can be seen with deep sedation. In these circumstances, so-called metabolic autoregulation is the cause of matched reduction of CBF, in the absence of ischemia. Conversely, when CBF reduction is greater than CMRO_2_ decrease, CBF becomes inadequate to satisfy energy demand, exposing the tissue to hypoperfusion and eventually ischemia. Importantly, low CBF and ischemia might occur despite CPP ≈ 65-75 mmHg [8]. Hence, CPP is not reliable to assess CBF in individual patients and other bedside tools are necessary.

**Figure 2:**
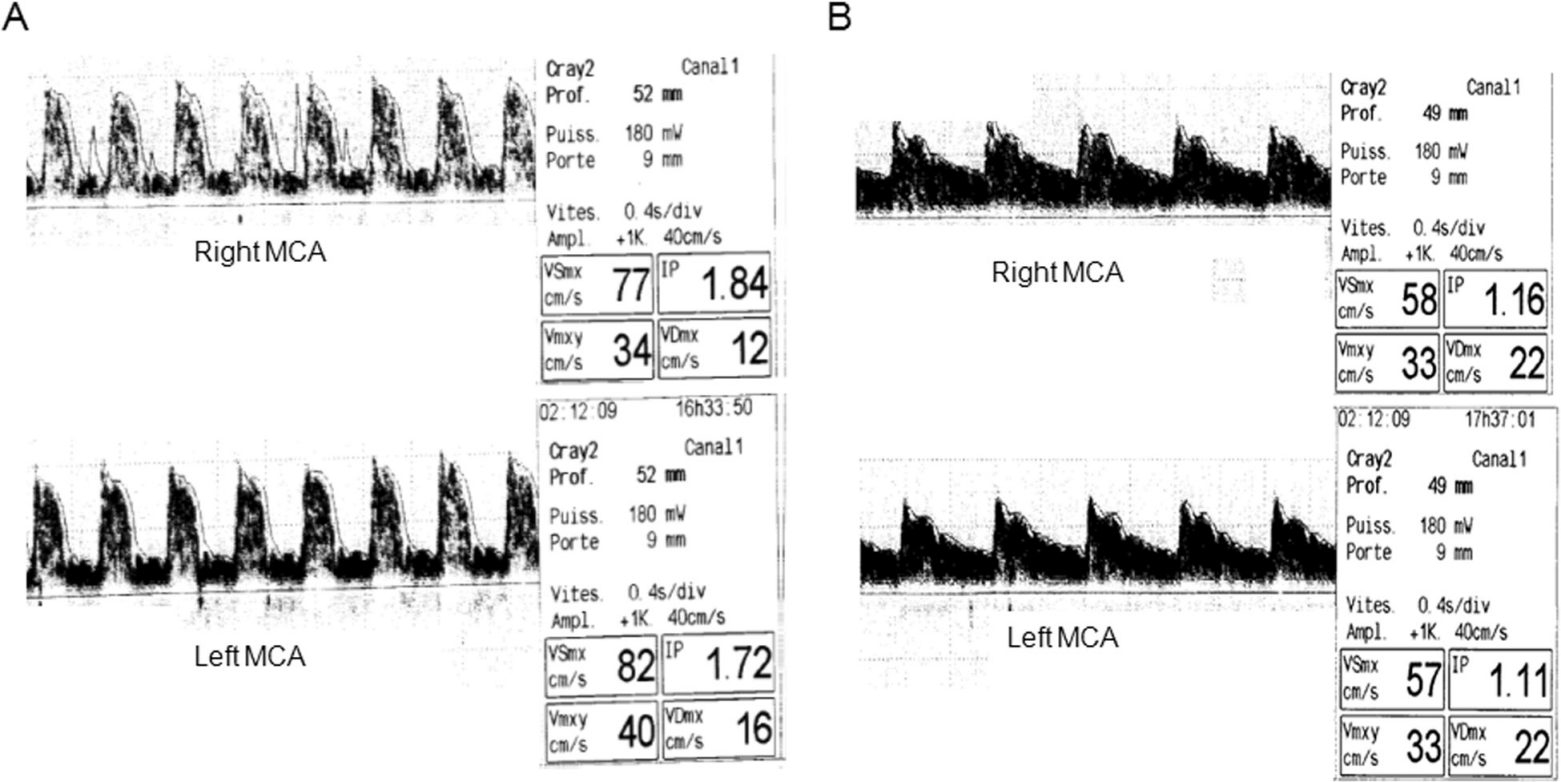
**Noninvasive transcranial Doppler to manage CBF/CPP at the bedside.** Example of transcranial Doppler in a patient with acute hydrocephalus and increased intracranial pressure (ICP). **A**. Before extraventricular drainage, TCD in the middle cerebral artery (MCA) shows cerebral ischemia with low diastolic CBF velocities (<20 cm/sec) and elevated pulsatility index (PI > 1.4). **B**. After extraventricular drainage, normalization of ICP was associated with normalization of diastolic velocities and PI, reflecting increased CBF.

Among noninvasive methods, TCD has been most studied and is an accurate tool to assess brain perfusion at the bedside. The technique consists in the measure of middle cerebral artery CBF velocities (CBFV; systolic, diastolic and mean) and the calculation of the pulsatility index [PI = (systolic CBFV – diastolic CBFV) / mean CBFV]. Reduced CBF, e.g., because of elevated ICP or low PaCO_2_, is diagnosed by low diastolic CBFV, a peaked waveform, and an elevated PI > 1.2-1.3 (Figure 2A). TCD has recently been used in the emergency room to detect high ICP/low CBF in TBI patients [9].

Invasive thermal diffusion flowmetry (TDF) is based on the insertion of an intraparenchymal probe (Thermal Diffusion Probe; Hemedex^®^ Cambridge, MA) generally next to ICP/PbtO_2_ probes. The technique uses thermal conductivity of the brain tissue and allows measuring regional cerebral blood flow (rCBF) in a quantitative way. The TDF technique was used in TBI to assess cerebral autoregulation and CO_2_ vasoreactivity and to calculate local cerebral vascular resistance. However, limitations of TDF, such as fever, sensor displacement, and the small area monitored, hindered its clinical application. Vajkoczy et al. showed good agreement between TDF and xenon-CT for regional CBF measurements [10]. Recent studies showed the potential utility of TDF, in combination with PbtO_2_, to optimize the management of CPP in brain-injured patients. In SAH patients, Muench et al. used TDF to guide medical therapy of delayed cerebral ischemia [11] and show that MAP augmentation was the only single useful intervention to improve CBF and PbtO_2_, whilst hypervolemia and hemodilution had only marginal effects at best, supporting recommended practices of induced hypertension alone over “triple-H” therapy for the medical management of delayed cerebral ischemia. After TBI, TDF may guide CPP management at the bedside and identify individual MAP/CPP thresholds [12]. Precise quantification of rCBF with TDF remains highly dependent on stable patient temperature and may be altered significantly in conditions of severe hyperthermia and rapid fluctuations of patient’s temperature. TDF seems a promising tool that may progressively become part of brain multimodality monitoring in the future. However, so far data are limited to small single-centre studies.

### Microvascular dysfunction

Macrovascular disturbances and reduced/inadequate CBF are not the only determinants of secondary ischemia. Impairment of the microvascular circulation also could play a key role in the constitution of secondary brain damages. Evidence for post-TBI microcirculatory dysfunction is both experimental and clinical. Marked disturbances in microcirculatory blood flow can be due to swelling of astroglial foot processes and compression of surrounding capillaries [13-15], or the formation of thrombi in the cerebral microcirculation [16]. In addition, electron microscopy showed endothelial edema, vacuolization and pinocytic vesicles [13,14,17]. Microvascular edema might cause increased barriers for oxygen diffusion with reduction of cellular oxygen delivery despite the absence of frank cerebral ischemia [17]. Microvascular damage accounts for the inability of pericontusional tissue to increase the oxygen extraction fraction (OEF = SaO_2_-SvO_2_/SaO_2_) in response to reduction of CBF induced by hyperventilation. Considering capillaries architecture as a central role for oxygen delivery to the cell [18], heterogeneity of red blood cell transit time into capillaries could lead to hypoxia in the injured brain, despite normal CBF.

The direct measurement of PbtO_2_ is now increasingly recognized as part of the bedside neuromonitoring after severe TBI [19] and can be used to detect brain hypoxia. Low PbtO_2_ has been reported to be associated with worse outcome after TBI [20,21], independently of brain hypoperfusion detected by ICP/CPP monitoring [22].

Laser Doppler flowmetry (LDF) is an invasive fiber-optic laser probe that can be inserted into brain parenchyma to measure regional perfusion of a tissue volume of approximately 1 mm^3^. Contrary to TDF, LDF provides only qualitative—but not quantitative—measurements of microvascular perfusion [23]. Routine use of this optical technique remains complex and artifacts, such as heterogeneity of microvascular architecture, probe motion, room temperature, and strong external light and sound may compromise data quality [24].

### Energy dysfunction

Brain activation and any augmentation of energy demand are followed by an increase in CBF and OEF. What is peculiar to the brain is its “avidity” for glucose utilization. Raichle and colleagues demonstrated that neuronal activation was followed by an increase in CBF and glucose utilization (≈30%) *without* a proportionate increase of OEF and cerebral metabolic rate of oxygen (CMRO_2_; ≈6%): this phenomenon is known as brain metabolic *uncoupling*[25]. Experimental and clinical studies have shown that glucose utilization may increase dramatically after TBI, in the absence of oxygen or CBF limitation (*cerebral hyperglycolysis*) [26-28]. This may lead to a reduction of cerebral glucose below the critical level and to a state of brain energy dysfunction or crisis [28,29]. In addition, cerebral oxidative metabolism may also be impaired due to mitochondrial dysfunction, thereby resulting in reduction of CMRO_2_ up to 50% in the acute period after TBI [30]. Altogether these findings support the view that nonischemic mechanisms are implicated in the pathophysiology of TBI.

## Bedside clinical approach

### Cerebral blood flow optimization

#### Determining the optimal CPP

Despite its limitations, the measure of ICP for the calculation of CPP is essential to the management of TBI at the bedside. Low CPP was well correlated with poor outcome in several studies [31,32]. All of these studies determined a critical ischemic threshold of CPP between 50 and 60 mmHg. Accordingly, and due to lack of benefit in increasing CPP to higher levels [33], actual recommendations from the Brain Trauma Foundation suggest a target of CPP between 50 and 70 mmHg [34]. However, recent clinical studies in TBI patients have repeatedly demonstrated that the so-called “optimal” CPP (i.e., the CPP threshold below which secondary ischemia occurs) differs individually and might vary over time within each single patient [6,8,35]. Arterial blood pressure/intracranial pressure (ICP)-derived pressure reactivity index (PRx) could be used to assess cerebrovascular pressure reactivity [36]. This index relies on spontaneous changes of arterial blood pressure and is calculated using a time correlation method. PRx, when averaged over specific CPP thresholds, demonstrated a U-shaped curve suggesting a specific relationship with the level of CPP. Although its utility can be debated, PRx may be useful in determining optimal CPP in individual patients. The reader also can refer to recent reviews on this particular topic [37].

Bedside measurement of PbtO_2_ also can be used as a surrogate marker of CBF. In agreement with recent data indicating that the venous fraction within cortical microvasculature exceeds 70%, it is suggested that PbtO_2_ predominantly reflects venous PO_2_[38]. Among the factors affecting PbtO_2_, the effect of decreased cerebral perfusion pressure (CPP) and CBF has been the most studied [39]. PbtO_2_ appears to correlate well with regional CBF and the relationship follows the autoregulation curve regulating CBF along a wide range of mean arterial pressure (MAP) [40]. The fact that increase in PbtO_2_ can be obtained with CPP and MAP augmentation further supports the notion that PbtO_2_ is a good marker of CBF and cerebral ischemia in certain conditions. Rosenthal and colleagues, however, using parenchymal TDP and PbtO_2_ monitoring, showed that PbtO_2_ more appropriately reflects the product of CBF and arteriovenous oxygen tension difference (AVTO_2_) [41]. Based on the formula PbtO_2_ = CBF ∙ AVTO_2_, reduced PbtO_2_ occurs frequently because of low CBF. However, PaO_2_ also is an important determinant of PbtO_2_[42] and additional pathological events (e.g., impaired lung function [43] or impaired O_2_ extraction due to increased gradients for oxygen diffusion in injured brain tissue [17]) might reduce PbtO_2_, in the absence of reduced CBF. Hemoglobin concentration also affects PbtO_2_ and reduced hemoglobin concentration below 9 g/dl may aggravate brain hypoxia [44].

**Figure 3:**
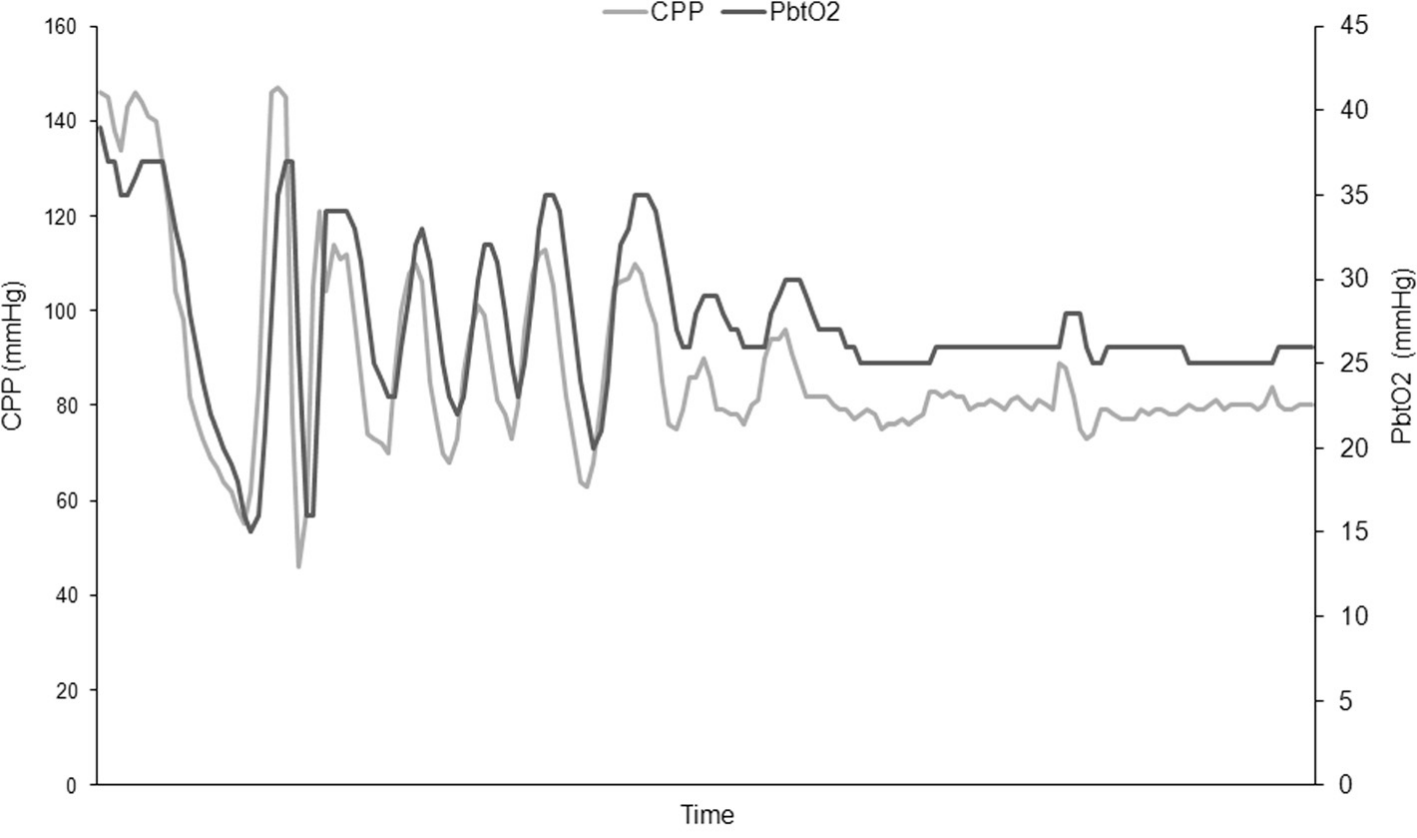
**PbtO_2_-guided management of CPP in individual patients.** Example of a patient exhibiting a linear correlation between CPP and PbtO_2_, which suggests impaired cerebrovascular reactivity (elevated oxygen reactivity index, ORx, > 0.7). In this case, higher CPP thresholds (>80 mmHg) are required to prevent secondary ischemia (PbtO_2_ < 20 mmHg). This is an example of how PbtO_2_ monitoring may guide CPP management and the setting of “optimal” CPP at the bedside.

**Figure 4:**
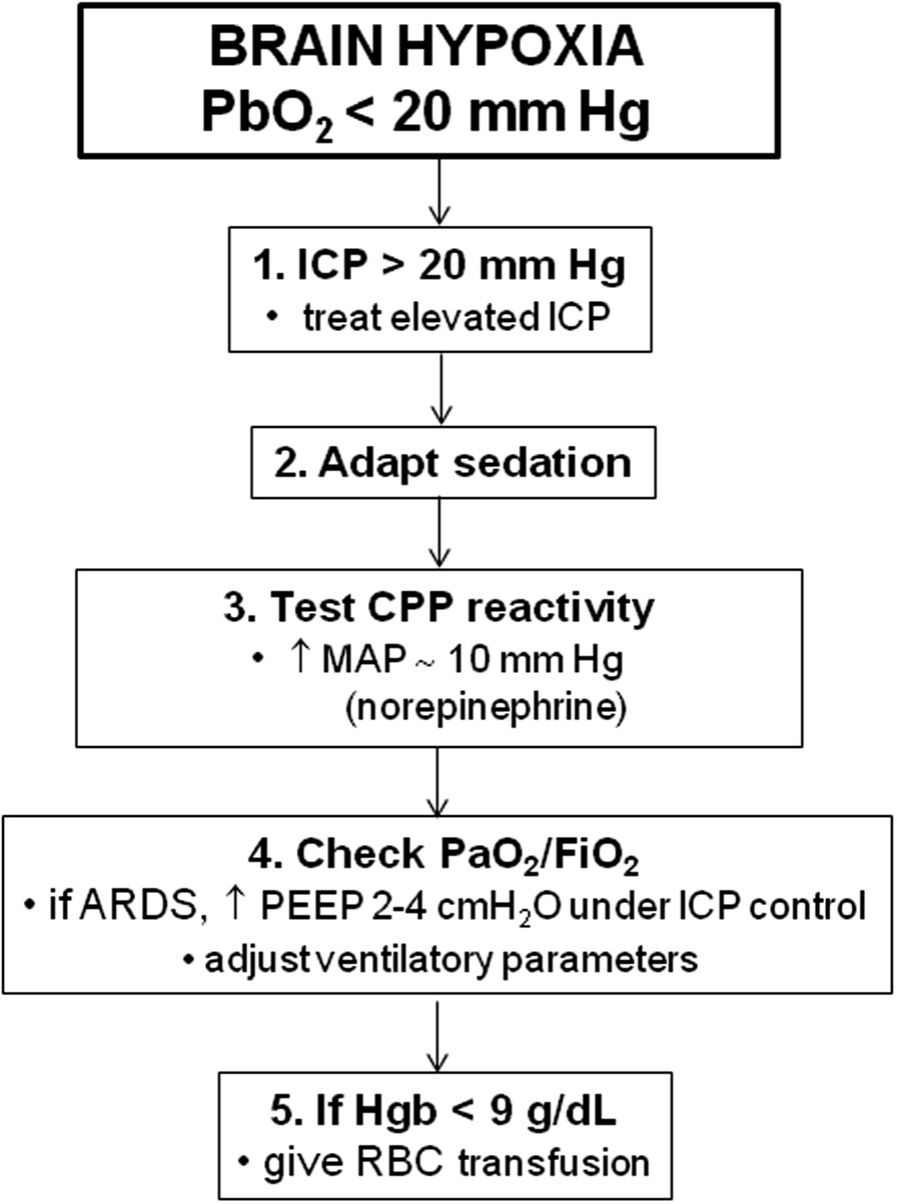
**Management of brain hypoxia.** A proposed algorithm for the practical management of low PbtO_2_ in patients with severe TBI.

CBF being an important determinant of PbtO_2_, it is possible to test at the bedside the individual response of PbtO_2_ to a vasopressor-induced increase of CPP/mean arterial pressure (MAP). This has originally been described as the *oxygen reactivity index* (ORx) and can be used to assess the state of cerebral autoregulation [45]. In practice, the PbtO_2_ response can be used to guide the management of CPP at the bedside. In pathological situations, the relationship between PbtO_2_ and CPP may become linear (Figure 3), hence manipulating CPP to maintain PbtO_2_ > 15–20 mmHg (PbtO_2_-directed strategy) might optimize CBF and avoid secondary ischemia [21,46]. Additional therapeutic interventions that may improve PbtO_2_ are described in section III.2 and Figure 4.

Measuring CBF velocities with TCD also could help physicians to manage CPP. A PI above 1.4 and a diastolic CBFV below 20 cm.sec^-1^ are characteristic TCD markers of ischemia after severe TBI [47], as illustrated in Figure 2B. In centers with clinical expertise, TCD is increasingly used to assess brain compliance, CPP and impending cerebral ischemia in patients in whom invasive ICP is not available. For example, TCD has been used in the early phase in the emergency room to detect brain ischemia non-invasively in TBI patients [47], before ICP monitoring was inserted. TCD also can be used to test brain autoregulation and vasoreactivity to CO_2_[36].

#### PaCO_2_

A reduction in PaCO_2_ results in a proportional reduction of CBF and cerebral blood volume. While this could be temporarily useful in decreasing elevated ICP, it must be underlined that prolonged aggressive hyperventilation (PaCO_2_ < 25–30 mmHg) is deleterious for patients with TBI by decreasing CBF below the ischemic threshold [48,49]. Regular measurements of PaCO_2_ with arterial blood gas analysis and continuous monitoring of end-tidal CO_2_ (EtCO_2_) must be achieved in every TBI patients to manage CBF variations secondary to PaCO_2_. Again, PbtO_2_ can be used to manage PaCO_2_ at the bedside [41,50]. Using CO_2_ reactivity, TCD also detects brain perfusion changes related to PaCO_2_ noninvasively and may be used to evaluate cerebral vasoreactivity and to tailor individual PaCO_2_ after TBI [51].

### Brain oxygen supply

Cerebral blood flow is one of the most important determinants of brain oxygen delivery. As discussed previously, arterial blood pressure and CPP are the major modifiable variables of brain oxygenation. This implicates that manipulating blood pressure and CPP may be the first and often the most effective intervention to optimize CBF and oxygen supply to injured brain tissue. Second-tier interventions to improve PbtO_2_ despite CPP modifications include optimization of systemic oxygenation (lung protective ventilation and maintenance of strict normoxia) [43] and red blood cell transfusion if hemoglobin is below 9 g/dl [44] (Figure 4).

However, other mechanisms may reduce brain tissue oxygenation. Among these, diffusion-limited oxygen delivery plays a key role and might explain why PbtO_2_ can be reduced despite oxygen delivery (PvO_2_) and CBF are normal [17]. In clinical practice, this may explain why in some circumstances brain tissue hypoxia can occur despite ICP/CPP being within normal ranges [22]. Without anemia or hypoxia, low PbtO_2_ despite adequate CBF probably reflects microcirculatory dysfunction and pericapillary edema [17]. This has important implications for the management of PbtO_2_ and the response of PbtO_2_ to therapeutic interventions.

#### Hyperoxia

One strategy to force diffusion barriers is to increase the fraction of dissolved oxygen with hyperoxia. All studies have demonstrated a robust effect of increasing FiO_2_ on brain oxygenation [52]. Whether this increase of PbtO_2_ is beneficial for patients remains controversial, probably due to the ability of the brain to use oxygen, i.e., the oxidative metabolism. Therefore, hyperoxia could benefit some patients in distinct cerebral areas [42]. However, considering the absence of strong evidence, this strategy is not recommended in TBI patients.

#### Erythropoietin

Erythropoietin (Epo) is a promising neuroprotective treatment in experimental models of TBI [53] and exerts significant cerebral antiedematous effect [54]. In an experimental model of diffuse TBI, it was recently shown that Epo, administrated intravenously up to 30 minutes after TBI, not only reverses cerebral edema but also significantly restores brain tissue oxygenation to normal levels [55]. Electronic microscopy revealed a decrease of astrocytic end-foot swelling, which could improve red blood cell transit time. Hence, Epo, given its combined effects on brain edema, perfusion, and oxygenation may be particularly promising for the treatment of TBI. The ongoing “Epo-TBI” study will clarify therapeutic potentials of Epo in patients with TBI (clinialtrials.gov: NCT00987454).

### Brain energy supply

Cerebral microdialysis (CMD) has largely contributed to a better understanding of the pathophysiology of acute brain dysfunction after TBI and was introduced recently as an additional bedside neuromonitoring tool in this context. CMD consists in the placement of an intraparenchymal probe that has on its tip a semi-permeable dialysis membrane. A cerebrospinal fluid-like solution, infused through this catheter, allows hourly sampling of patients’ brain extracellular fluid into microvials for bedside analysis [56]. CMD provides continuous monitoring of cerebral energy metabolism at the bedside and the measurement of absolute and dynamic changes of brain extracellular concentrations of glucose, pyruvate, and lactate. Additional markers, such as glutamate (excitotoxicity) and glycerol (membrane integrity), can be measured [57,58]. The clinical utility of CMD is mainly to detect impending ischemia/hypoxia and to assess the energetic state of the injured human brain [19,59,60]. Abnormal lactate/pyruvate ratio (LPR) is defined by a LPR > 25 [56], and this threshold is associated with worse outcome after TBI [57]. Others also used LPR > 40 as marker of cell energy crisis [56]. In clinical practice, values >35-40 are used to start therapeutic interventions. Elevated LPR can be due to ischemia/hypoxia: in these circumstances, elevated lactate is accompanied by low pyruvate, as well as low cerebral glucose and brain oxygen. “Ischemic/hypoxic” LPR elevations can reach very high levels, well above 40. A second pattern also can be seen, where elevated lactate/normal-to-high pyruvate is seen and indicates the cause of LPR elevation is nonischemic in nature and can be due to hyperglycolysis [61] or mitochondrial dysfunction [28]. In this second “nonischemic” pattern levels of LPR are often only slightly elevated (LPR 40–50). Elevated LPR is therefore not only a marker of ischemia, but rather reflects the metabolic state of the tissue.

#### Brain glucose supply and blood glucose control

**Figure 5:**
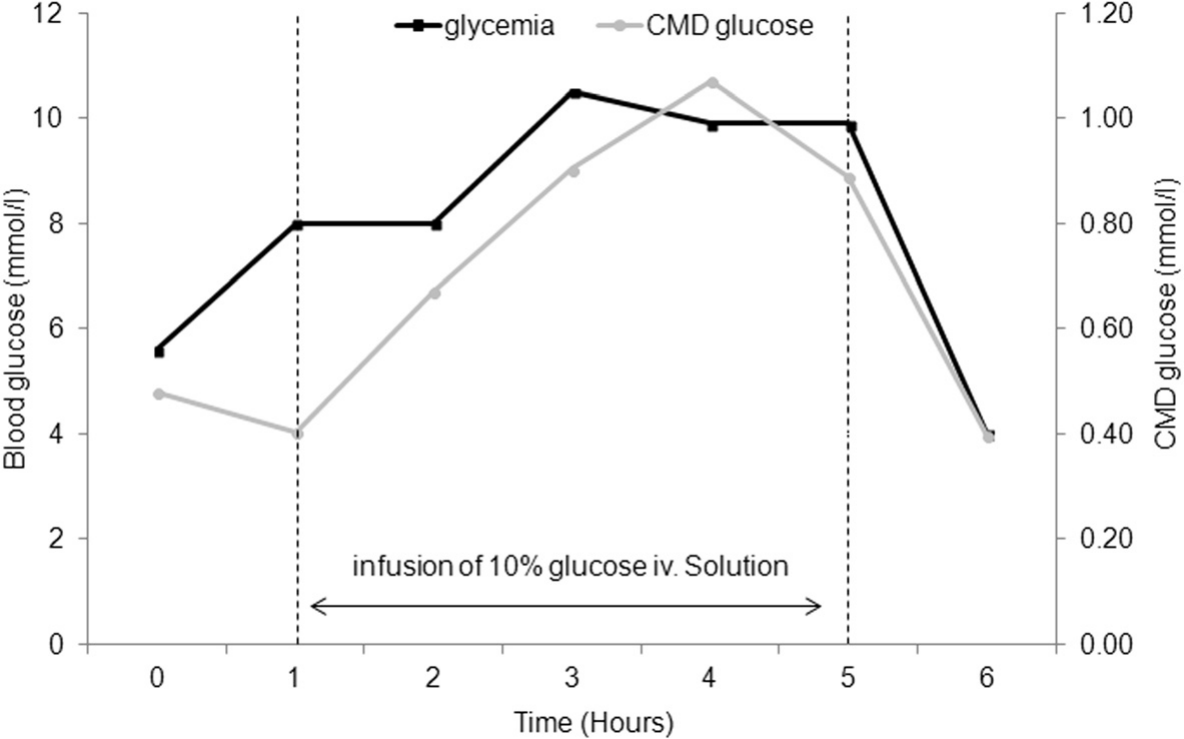
**Cerebral microdialysis-guided management of glycemic control in individual patients.** Example of a patient showing a linear relationship between blood and brain glucose, measured by cerebral microdialysis (CMD) glucose. Because of low CMD glucose <1 mmol/L, infusion of a 10% glucose solution was administered and was associated with a parallel increase of both arterial blood and CMD glucose. This illustrates the potential value of CMD for the management of blood glucose control in patients with severe brain injuries, aiming to prevent secondary systemic insults (brain glucopenia in this case).

Cerebral microdialysis has greatly contributed to better manage glucose control in TBI patients at high risk for secondary brain injury [62,63]. Glucose is the main energy source for the human brain. Therefore adequate glucose supply is crucial to maintain brain function. Supply of glucose is provided by selective transporters (GLUT) that allow glucose diffusion across the blood brain barrier to brain cells. Glucose supply to the brain is highly dependent on the availability of glucose from the systemic circulation (Figure 5) [64]. Therefore, so-called “intensive” blood glucose control with the use of insulin therapy may reduce brain glucose availability and potentially increase energy dysfunction or aggravate metabolic distress [63,65-67]. Moderate (≈8-10 mmol/L) vs. intensive (≈4.5-6 mmol/L) glucose control does not confer any benefit on TBI outcome [68]. Using CMD, systemic glucose concentration can be targeted to CMD glucose, to avoid neuroglucopenia (CMD <1 mmol/L). In some patients, this can occur already at blood glucose levels <8 mmol/l, and therefore systemic glucose must be adapted to avoid low brain glucose, by keeping blood glucose at 8–10 mmol/L, if necessary by giving slow infusion of 10% i.v. glucose. It is important to realize however that reduced brain glucose can be due to other reasons, including ischemia/energy crisis, elevated ICP/low CPP; in these circumstances, these causes must be treated first. Given these findings, when CMD is available precise blood glucose levels should be targeted to CMD glucose to avoid levels <1 mmol/L. Otherwise, moderate blood glucose control (≈8-10 mmol/L) is recommended for the management of patients with severe brain injury.

#### Alternative energy substrates

Glucose utilization involves two different pathways. Oxidative phosphorylation is the energy-generating biochemical process whereby pyruvate, produced by glycolysis, is oxidized to CO_2_ and H_2_O with the production of 30 moles of ATP. This process requires oxygen. Glycolysis is the energy-generating biochemical process whereby glucose is converted to pyruvate and lactate with the net production of 2 moles of ATP. This process is nonoxidative, producing lactate [25].

Evidence from *in vitro* and *in vivo* studies demonstrates that lactate is an important energy substrate for neurons [69], particularly in conditions of hypoxia [70]. Recently, evidence of brain lactate utilization in humans with acute brain injury has been suggested [61,71]. Exogenous administration of lactate with the use of sodium lactate perfusions improves cerebral performance during intense exercise [72] and in diabetic patients [73], with sparing of brain glucose [74]. Preliminary studies in TBI patients suggest that sodium lactate solutions may be a future therapeutic strategy, potentially more effective to lower ICP than mannitol [75].

## Conclusions

ICP/CPP monitoring is important after TBI since CBF is highly dependent on CPP below the lower limit of cerebral autoregulation, i.e., CPP <50 mmHg. Above the ischemic threshold, tailoring CPP for each patient needs a more comprehensive approach guided to brain multimodal monitoring to target CBF, oxygen delivery, and supply of brain energy substrates individually. Intraparenchymal PbtO_2_ reflects the complex interaction between CBF and oxygen delivery/consumption by the injured brain. As a result, PbtO_2_-targeted therapy might help managing CPP and prevent secondary cerebral ischemia and can be considered as a useful addition to standard ICP monitoring. Transcranial Doppler also can help to diagnose reduced CBF and elevated ICP at the bedside noninvasively and may help to direct in the early phase the need for additional intracranial monitoring and for emergent surgical interventions. Cerebral microdialysis provides essential information on brain metabolism and the availability of main energy substrates (mainly glucose) and also is potentially useful to detect secondary delayed cerebral ischemia and manage blood glucose control. Beyond ICP monitoring, contemporary management of severe TBI patients is increasingly based upon a more comprehensive clinical approach that must not be limited to ICP/CPP therapy but also includes the individual optimization of CBF, oxygen, and energy substrate delivery guided by bedside brain multimodal monitoring.

## Abbreviations

ATP: Adenosine tri-phosphate; CBF: Cerebral blood flow; CBFV: Cerebral blood flow velocity; CBV: Cerebral blood volume; CMD: Cerebral microdialysis; CMRO2: Cerebral metabolic rate of oxygen; CO2: Carbon dioxide; CPP: Cerebral perfusion pressure; CT: Computed tomography; Epo: Erythropoietin; H2O: Dihydrogen oxide; ICP: Intracranial pressure; LDF: Laser Doppler flowmetry; LPR: Lactate/pyruvate ratio; MCA: Mild cerebral artery; MRI: Magnetic resonance imaging; MTT: Mean transit time; O2: Oxygen; OEF: Oxygen extraction fraction; PaO2: Arterial partial pressure of oxygen; PbtO2: Brain tissue oxygenation pressure; PET: Positron emission tomography; PI: Pulsatility index; SAH: Subarachnoid hemorrhage; TBI: Traumatic brain injury; TCD: Transcranial Doppler; TDF: Thermal diffusion flowmetry.

## Competing interests

The author’s declare that they have no competing interests.

## Authors’ contributions

PB, NS, JFP and MO drafted the manuscript. All authors read and approved the final version.
